# Neuropsychiatric disorders: post-stroke depression. A review

**DOI:** 10.1192/j.eurpsy.2025.1345

**Published:** 2025-08-26

**Authors:** M. Martínez Grimal, J. J. Tascón Cervera, A. M. Morales Rivero, F. A. Rodríguez Batista, E. E. Morales Castellano

**Affiliations:** 1 Hospital Universitario de Gran Canaria Doctor Negrín, Las Palmas de Gran Canaria, Spain

## Abstract

**Introduction:**

Post-stroke depression (PSD) is the most common neuropsychiatric disorder after stroke, and also the main factor limiting recovery and rehabilitation in stroke patients, which leaded to poor quality of life. A prevalence of 30% is estimated between 1 to 5 years after stroke. More than half of all cases are neither diagnosed nor treated.

**Objectives:**

The aim of this paper is to review the latest updates regarding post-stroke depression and progress in its diagnosis and treatment.

**Methods:**

A systematic review of the scientific and clinical literature on PSD was conducted. The review included databases such as PubMed and Cochrane, covering articles from the past 10 years. The scientific evidence obtained was analyzed and synthesized.

**Results:**

Currently, the diagnosis of PSD is mainly based on the DSM guidelines and combined with various depression scales. Symptoms usually occur within the first three months after stroke, the patient presents symptoms of a depressive episode such as low mood, anhedonia, loss of appetite, sleep disorders, vegetative symptoms or social withdrawal. Several mechanisms, including biological, behavioural, and social factors, are involved in its pathogenesis. The main predictors are personal history or cognitive impairment, as well as sequelae of the stroke. Left hemispheric strokes are those with the highest risk of early depression, as well as small subcortical vessel pathology. Treatment is mainly pharmacological, with SSRIs. The probability of recovery is between 15 and 57% in the first year, with a recurrence of 38% at two years, up to 100% at 15 years. In addition, there is an increase in mortality, especially in those under 65 years of age.

**Conclusions:**

There are still many unanswered questions in the treatment of PSD, such as the best time to start treatment or the effects of antidepressants on cognition and motor function, among others. Although great advance has been made by researchers, the mechanism of PSD is not completely clear.

**Disclosure of Interest:**

None Declared

